# Loneliness inside of the brain: evidence from a large dataset of resting-state fMRI in young adult

**DOI:** 10.1038/s41598-022-11724-5

**Published:** 2022-05-12

**Authors:** Denilson Brilliant T., Hikaru Takeuchi, Rui Nouchi, Ryoichi Yokoyama, Yuka Kotozaki, Seishu Nakagawa, Sugiko Hanawa, Atsushi Sekiguchi, Shigeyuki Ikeda, Kohei Sakaki, Kelssy Hitomi dos Santos Kawata, Takayuki Nozawa, Susumu Yokota, Daniele Magistro, Ryuta Kawashima

**Affiliations:** 1 Department of Advanced Brain Science, Institute of Development, Ageing and Cancer, Tohoku University, Sendai, Japan; 2 Division of Developmental Cognitive Neuroscience, Institute of Development, Ageing and Cancer, Tohoku University, Sendai, Japan; 3 Smart-Aging Research Center, Tohoku University, Sendai, Japan; 4 Departments of Cognitive Health Science, Institute of Development, Ageing and Cancer, Tohoku University, Sendai, Japan; 5 Suwa Red Cross Hospital, Suwa, Japan; 6 Division of Clinical Research, Medical-Industry Translational Research Center, Fukushima Medical University School of Medicine, Fukushima, Japan; 7 Department of Human Brain Science, Institute of Development, Ageing and Cancer, Tohoku University, Sendai, Japan; 8 Division of Psychiatry, Tohoku Medical and Pharmaceutical University, Sendai, Japan; 9 Department of Behavioral Medicine, National Center of Neurology and Psychiatry, National Institute of Mental Health, Tokyo, Japan; 10 RIKEN Center for Advanced Intelligence Project, Tokyo, Japan; 11 Department of Mechanical and Intelligent Systems Engineering, The University of Electro-Communications, Tokyo, Japan; 12 Research Institute for the Earth Inclusive Sensing, Tokyo Institute of Technology, Tokyo, Japan; 13 Faculty of Arts and Science, Kyushu University, Fukuoka, Japan; 14 Department of Sport Science, Nottingham Trent University, Nottingham, England

**Keywords:** Neuroscience, Psychology, Neurology

## Abstract

Although loneliness itself is a natural emotion, prolonged loneliness is detrimental to human health. Despite its detrimental effect, few loneliness-related neuroimaging studies have been published and some have limitations on the sample size number. This study aims to find the difference in resting-state functional connectivity associated with loneliness within a big sample size via the seed-based approach. Functional connectivity analysis was performed on a large cohort of young adults (*N* = 1336) using the seed-based functional connectivity approach to address the concern from previous studies. The analysis yielded statistically significant positive correlations between loneliness and functional connectivities between the inferior frontal gyrus and supplementary motor area, precentral gyrus, and superior parietal lobule. Additionally, the analysis replicated a finding from a previous study, which is increased functional connectivities between the inferior frontal gyrus and supplementary motor area. In conclusion, greater loneliness is reflected by stronger functional connectivity of the visual attention brain area.

## Introduction

Loneliness is defined as the subjective feeling of being socially isolated. Loneliness itself is different from being alone; a person can feel happy and not distressed alone, while the same person can also feel lonely and distressed despite being surrounded by many people another time. Although loneliness is a common experience across all age^1^, it is specifically highly prevalent in the young adult population^2^. The prevalence is concerning as the young adult period is defined as the transitional period of the life course that a decision taken in this period will strongly affect long-term life trajectories^3^. Moreover, loneliness is also associated with poorer human health, specifically mental health^4^.

A model of loneliness proposed by Cacioppo and Hawkley in 2010 proposes that due to the social pain inflicted from perceived social isolation, lonely individuals exert unconscious surveillance for potential social threats labeled as implicit hypervigilance. The implicit hypervigilance consists of attentional bias and confirmatory behavior that cause the individuals to view the social world as threatening leading to expecting and displaying negative social behavior and affect^4,5^. Attentional bias refers to the increased attention toward negative social information that potentially causes rejection^6^. Confirmatory behavior consists of inappropriate social and withdrawal behaviors that could elicit negative responses and rejection from others, thus, confirming the initial negative belief about the interaction^5,7^. Consequently, these manifestations of negative social interaction trigger perceived social isolation feeling repeating the cycle^4^.

Neural correlates may explain the underlying mechanism why the behaviors related to loneliness manifest. Previously, structural magnetic resonance imaging (MRI) studies demonstrated that loneliness is associated with smaller white matter structures in general^8,9^ and higher and lower grey matter volumes depending on the area^10–12^. Moreover, functional MRI (fMRI) studies also demonstrated that loneliness is associated with differences in brain activation^13–15^. However, although many studies show that loneliness is correlated with brain structures and functions^8–16^, limited studies have been performed on the resting-state functional connectivity related to loneliness^17–20^. Applying functional connectivity analysis during a resting state can reveal brain intrinsic organization and information processing^21^. Therefore, applying the resting-state fMRI approach on loneliness will be appropriate to find the intrinsic brain organization.

As previously stated, the hypervigilance of loneliness consists of attentional and confirmatory bias^4^. Both studies by Layden et al. and Tian et al. in 2017 support the attentional bias of hypervigilance. Increased functional connectivity among cinguloopercular network areas, such as inferior frontal gyrus (IFG) and supplementary motor area (SMA)^17^, and lower causal flow of dorsal to ventral attention network, such as superior parietal lobule (SPL) to IFG^20^, may contribute to the increased social monitoring for potential social threat. However, despite supporting the theory, those previous studies have a limited sample size, which is less than a hundred. Although a small sample size can be a good starting point for a study^22^, those studies may overestimate the magnitude of the findings and become surrogate markers. These results may have a relationship but not a guaranteed one^23^. Certainly, this limitation has been addressed as a critical concern according to the recent review of fMRI studies^24^. To resolve the limitation, the current study recruited a large number of participants. Therefore, this study is a conceptual replication of prior research to confirm the relationship between loneliness and functional connectivity among the visual attention areas.

On the other hand, studies by Mwilambwe-Tshilobo et al. in 2019 and Spreng et al. in 2020 tackled the sample size limitation. Both studies used the independent component analysis (ICA) approach to construct the resting-state network. Although the ICA approach is less prone to noise and does not need a prior assumption, it suffers the disadvantages of run-to-run variability on the resting-state network due to iterative optimization and arbitrary model order^25^. Considering the disadvantages of the ICA approach and the prior assumption on visual attention areas, the seed-based approach would be used to construct the resting-state network. Moreover, both studies also supported the confirmatory behavior of loneliness. Increased functional connectivity density between default mode and frontoparietal network areas may contribute to prolonged negative affect^18^ while increased functional connectivity among default mode network areas may be suggested for increased mental simulations of social events with distorted social functioning^19^. Negative affect and distorted mental simulation are some examples of confirmatory behaviors of loneliness^5^. For these reasons, social functioning areas, such as dorsomedial prefrontal cortex (DMPFC)^26^ and temporoparietal junction (TPJ)^27^, become the secondary interest of this study.

The objective of this study is to find the difference in resting-state functional connectivity related to loneliness within a large sample size. Moreover, this study is a conceptual replication to ensure the replicability of the previous studies’ findings. The hypotheses of this study are: (1) positive correlations between loneliness score and functional connectivity among IFG, SMA, and SPL; (2) a negative correlation between loneliness score and functional connectivity between DMPFC seed and TPJ will be observed. The mentioned brain areas play a role in visual attention^28–30^ and social functioning^26,27^ respectively. These results are expected due to the previous studies^6,14,15^ and the neural finding of the previous resting-state functional connectivity study^17–20^ suggesting that loneliness is associated with increased monitoring and inappropriate social behavior. This study is a conceptual replication in a large sample to confirm the functional connectivity related to visual attention and social brain areas. Therefore, this is the first study that observes the neural correlates of loneliness using the seed-based approach in a large young adult population.

## Results

### Descriptive result

267 participants data were excluded from all participants due to excessive head motion shown by framewise displacement (FD) Power greater than 0.20 leaving 1336 eligible participants data (F:M = 581:755) for this study analysis. The data with FD value greater than 0.20 are excluded to maximize the removal of motion artifacts and minimize the removal of non-motion signals^31^. For statistical analysis purposes, females were coded as 1 and males as 0. The descriptive statistics of age, loneliness score, and Raven’s advanced progressive matrices test score (RAPMT) of eligible participants are shown in Table 1. RAPMT was used to assess the general intelligence level to rule out the general intelligence effect on resting-state fMRI measures^32^. The Pearson’s correlation coefficients among these measures are shown in Table 2.

**Table 1:** Descriptive statistics.

	Lowest	Highest	Average	SD
Age	18	27	20.8	1.7
Loneliness	20	76	37.1	8.9
RAPMT	12	36	28.5	3.8

SD: standard deviation.

**Table 2:** Pearson’s correlation among descriptive statistics.

	Age	Sex	Loneliness	RAPMT
Age	1			
Sex	− .040	1		
Loneliness	− .031	− .132*	1	
RAPMT	− .005	− .093*	.066*	1

*p < .05; RAPMT: Raven’s advanced progressive matrices test.

**Figure 1:**
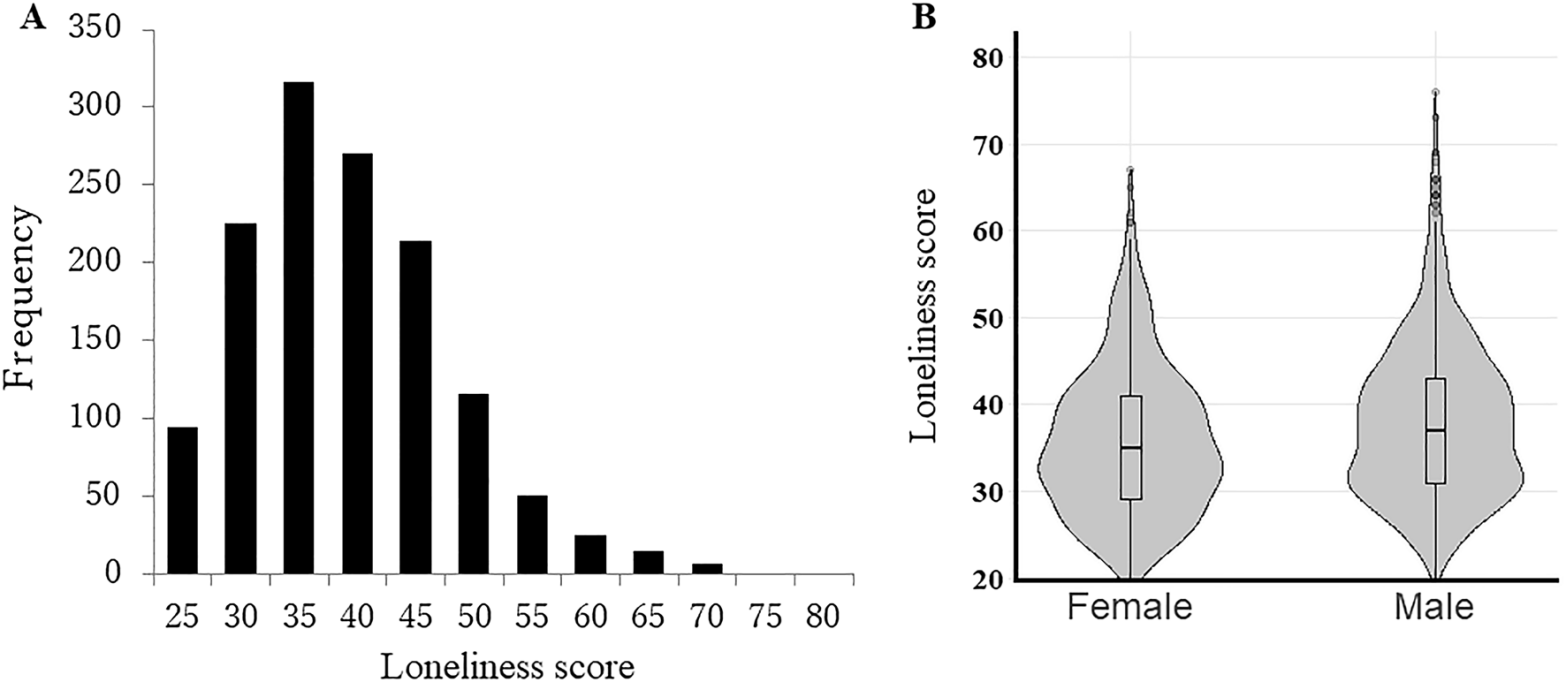
The distribution of loneliness among participants. (**A**) Overall distribution. (**B**) Female-male distribution.

The loneliness score of participants ranged from 20 to 76 with an average of 37.13 (SD = 8.92). The distribution of loneliness in the participants is positively skewed. The distribution of loneliness is shown in Fig. 1.

### Functional connectivity

Of the four connectivity seeds: anterior insula, DMPFC, IFG, and TPJ, used in the functional connectivity analysis, only two seeds yielded findings. The seeds are IFG and DMPFC, which are expected from the prior assumption of this study. After regressing out age, sex, RAPMT, and FD and undergoing 5000 permutations, a second-level multiple regression analysis between functional connectivity and loneliness yielded three significant positive correlations and a trend of negative correlation between resting-state functional connectivity and loneliness score was found. The table of functional connectivity findings is presented in Table 3.

**Table 3:** List of functional connectivity findings that are associated with the loneliness score.

Seed	Connectivity	Name	k	TFCE	*x*	*y*	*z*	*p*
IFG	Positive	SMA	38	299.10	0	− 15	64	.0277
		PreCG	10	276.54	− 23	− 19	79	.0427
		SPL	7	278.24	15	− 60	56	.0414
DMPFC	Negative	TPJ	7	250.31	− 49	− 45	34	.0645

k: voxel size, TFCE: Threshold-free cluster enhancement value; x,y,z: coordinates in MNI; DMPFC: Dorsomedial prefrontal cortex; IFG: Inferior frontal gyrus; PreCG: Precentral gyrus; SMA: Supplementary motor area; SPL: Superior parietal lobule; TPJ: Temporoparietal junction.

**Figure 2:**
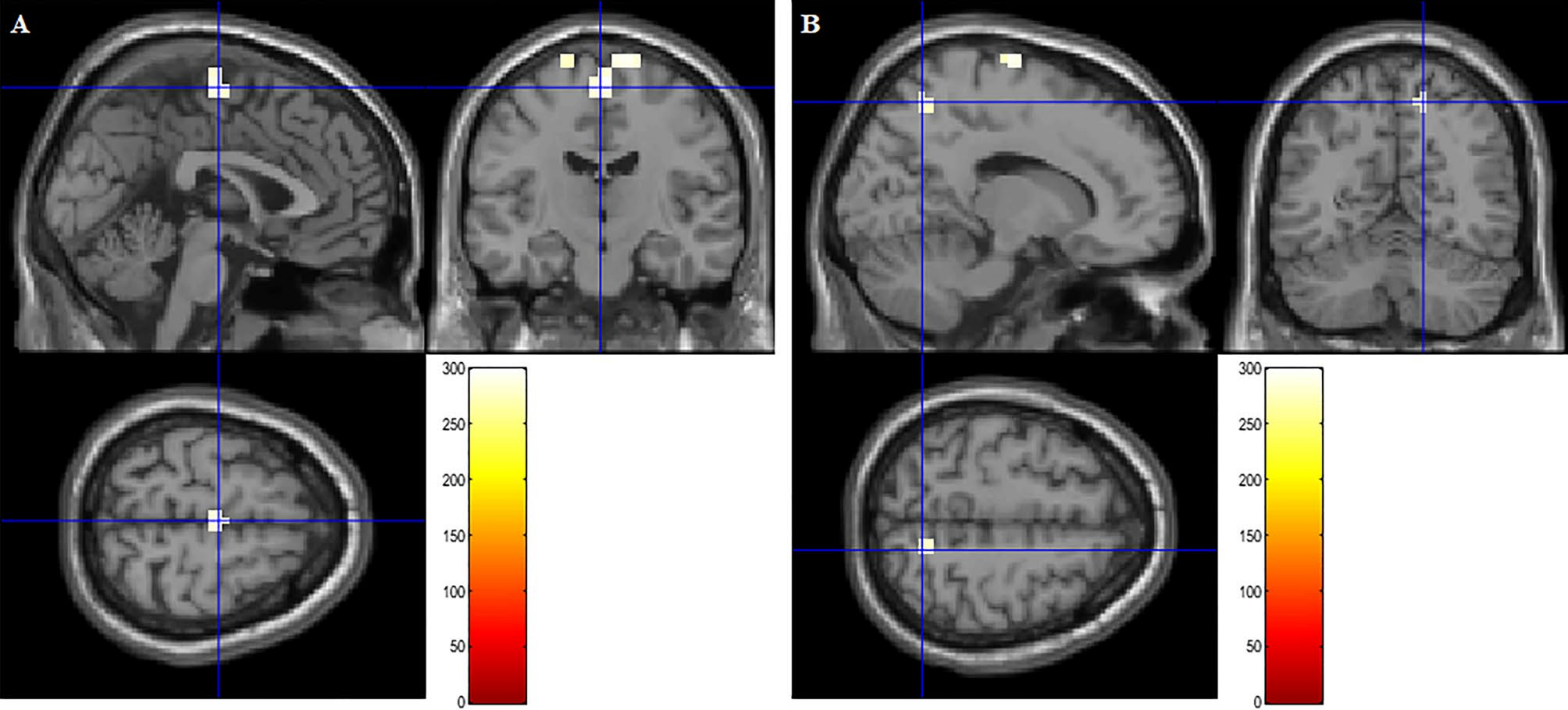
Significant positive effects of loneliness on resting-state functional connectivity with IFG as the seed region. The color represents the strength of the TFCE value. (**A**) The result was corrected within SMA. (**B**) The result was obtained using the TFCE of *p* < .05 based on 5,000 permutations. The result was corrected within the right SPL.

Multiple regression analysis between functional connectivity coefficient with IFG seed and loneliness score showed three significant increased functional connectivities. The functional connectivity between IFG and SMA (*x*, *y*, *z* = 0, − 15, 64; Threshold-free cluster enhancement (TFCE) = 299.10; *p* < 0.05 familywise error corrected (FWE)), IFG and precentral gyrus (PreCG) (*x*, *y*, *z* = − 23, -19, 79; TFCE = 276.54, *p* < 0.05 FWE), and IFG and SPL (*x*, *y*, *z* = 15, − 60, 56; TFCE = 278.24, *p* < 0.05 FWE) are positively correlated with loneliness score. These findings are consistent with the first hypothesis. Additionally, one of the findings replicated the previous study finding^17^. Functional connectivity between IFG and SMA is shown in Fig. 2A. Functional connectivity between IFG and PreCG and IFG and SPL are shown in the Fig. 2B.

Another multiple regression analysis between functional connectivity coefficient with DMPFC seed and loneliness score also showed a marginally significant decreased functional connectivity with loneliness score. This finding is also consistent with the second hypothesis, albeit reaching marginal significance. Moreover, this finding has not been found in the previous studies. Please see the Supplementary material.

## Discussion

This is the first large-scale neuroimaging study researching the resting state correlates of loneliness in the young adult population using the seed-based approach. The participants’ loneliness score was lower than a previous survey study on Japanese young adults to elderly^33^. Additionally, this study addresses the limitations of the previous functional connectivity study with the larger sample size.

This study investigated the association between functional connectivity and loneliness in the general population of young adults. The findings of this study match the hypotheses. Whole-brain analysis revealed four notable findings of functional connectivity associated with loneliness score, three increased functional connectivities and one decreased functional connectivity. Young adults with higher loneliness have stronger functional connectivity between seed area right IFG and SMA, PreCG, and SPL. Among these results, increased functional connectivity between right IFG and SMA is consistent with the previous study^17^. All increased functional connectivities reached the significant value FWE less than 0.05. On the other hand, the young adult with higher loneliness has weaker functional connectivity between DMPFC and left TPJ. However, the decreased functional connectivity reached the marginally significant value FWE less than 0.10. The current findings can update the findings of the previous functional connectivity study^17^.

The functional connectivity between the seed IFG and SMA extending to the right PreCG is significantly correlated with the loneliness score. IFG plays roles in executive control^34^, social cognition^35^, and selective and spatial attention^28,30^. On the other hand, SMA is activated during voluntary movement^36,37^ and temporal coordination in sequential movement^38^. Moreover, a part of SMA called the supplementary eye field plays a role in oculomotor control^38^. Increased functional connectivity between both areas may imply that lonely individuals are cautious about negative cues by actively observing their surroundings. The same finding is also found between the IFG and left PreCG. PreCG is the area of the primary motor cortex, which plays a role in voluntary planning^39^ and pain appraisal^40^. With actively observing their surroundings, it may also suggest that lonely individuals are also carefully planning and appraising potential threats against themselves.

The IFG and SMA functional connectivity was also observed in the previous study^17^. Layden et al. in 2017 performed functional connectivity of loneliness and depressive symptoms. The study suggested that increased functional connectivity between IFG and SMA may underlie the implicit hypervigilance of loneliness by sustained and maintained tonic alertness^17^. Implicit hypervigilance is a loneliness theory suggesting that lonely individuals see the social world as more threatening by exerting over-attention over negative social information^4,41^. Finding the same functional connectivity with the same seed indicates successful replication of the previous study’s finding. Moreover, the other findings also support the replicated finding.

Alternatively, increased functional connectivity between IFG and SMA may also be interpreted as the desire to reconnect with others. IFG is also an area for mirror neurons fundamental to human social interaction^42^. Dysfunction in the mirror system consequently leads to social skill deficits, similar to autism spectrum disorders and schizophrenia^43^. With oculomotor control, mirroring behavior may be carried out, hoping to increase affiliation and liking between people^44^. Therefore, the increased connectivity of IFG and SMA may potentially imply the elevated desire to reconnect with others by mirroring others’ behavior. However, this interpretation remains speculative and should be interpreted cautiously because no previous study showed similar findings related to social reconnection. A previous study showed the ventral tegmental area and orbitofrontal cortex play a role in social craving after long-hour of isolation^45^. Because of the difference between the loneliness state used as the condition in the previous study^45^ and the loneliness trait of this study, further research needs to be performed to confirm the speculation and the relation between social craving and desire to reconnect.

Another finding, increased functional connectivity between IFG and SPL is also observed. SPL plays roles in working memory^46^ and attention and visuospatial perception^29^. Together with the previously mentioned findings, this functional connectivity may reinforce the attention and threat monitoring of surroundings, thus, further enhancing the attention.

The implication of the trend of decreased functional connectivity between DMPFC and left TPJ is discussed in the supplementary discussion. Please see the Supplementary material.

Despite the strength of this paper on the sample size and replication of previous findings, there are some limitations found in this study. First, this study is a cross-sectional study. The findings of this study are observed at a time point. The progression of connectivity across periods is currently still unavailable. Longitudinal studies on loneliness are needed to better understand how loneliness develops, including the direction of causality between the neural organization and feeling lonely. Second, this study is observational. This study is not an intervention study that manipulates variables. All interpretations above are based on previous studies’ assumptions. Although it is tempting to use causation, correlation is not equal to causation. On the other hand, it will be difficult to conduct an intervention study for loneliness considering the ethical issue of the risk–benefit, which can be similar to Milgram’s experiment^47^. Therefore, the implication of the findings should be interpreted very cautiously. Third, loneliness assessment. The questionnaire of loneliness in this study was the UCLA loneliness scale due to the validity and reliability of the questionnaire^48^. Although its validity and reliability, UCLA only measures the overall loneliness. Recent studies showed that UCLA loneliness consists of multiple subcomponents depending on the criteria used for categorization^49,50^. Loneliness also manifests several factors, such as social vigilance, social avoidance, depressive mood, anxiety trait, and cognitive bias^4,51^. Future studies using behavior tasks or specific psychological measurements followed by loneliness assessment can understand loneliness better.

In conclusion, this study showed significant associations between resting-state functional connectivity and loneliness from a large dataset in the young-adult age category. Those associations are shown between right IFG seed and SMA, left preCG, and right SPL, and DMPFC seed and left TPJ. The associations between the right IFG seed and the respective brain regions were positive correlations. Moreover, the positive correlation between right IFG and SMA is consistent with the previous study. On the other hand, the association between DMPFC and left TPJ was observed as a negative correlation, albeit reaching marginal significance. These findings suggest that greater loneliness is reflected by significantly stronger functional connectivity among areas that play a role in visual attention and marginally significantly weaker functional connectivity between areas that play a role in social functioning.

## Methods

### Participant

The present study is a part of an ongoing project investigating the associations among brain imaging, cognitive function, and aging^52^. 1603 healthy right-handed participants (688 female, 915 males) were recruited. Those who had a history of neurological or psychiatric illness were excluded. The sex ratio difference of this study participants was due to the unbalanced sex ratio in the participant pool. The sex variable was dummy coded as one for females and zero for males. The mean age of participants is 20.77 (SD = 1.73, age-range = 18–27). All participants were undergraduate and post-graduate students at Tohoku University. Written informed consent was obtained from each participant in accordance with the Declaration of Helsinki (1991). The study was approved by the Ethics Committee of Tohoku University.

Participants were instructed to get sufficient sleep, maintain body fitness, and consume a normal diet, including caffeinated food and drinks, on the day of the study. However, Participants were instructed to avoid alcohol consumption the night before the study. The description in this subsection is mostly reproduced from our previous study^52,53^.

### Loneliness assessment

The revised University of California, Los Angeles (R-UCLA) Loneliness Scale version 3 was used to assess the perceptions of social isolation and loneliness. The questionnaire was proven as a consistent (Cronbach’s α: 0.89–0.94) and a reliable (test–retest *r*: 0.73) measure for loneliness over a two-month period^48^. It consists of 20 items rated from 1 (never) to 4 (often) resulting in the loneliness score ranging from 20 to 80. The higher the score the greater the loneliness. Some examples of the items are “How often do you feel that you lack companionship?” and “How often do you feel left out?”. In this study, the Japanese version of the questionnaire was used as some previous studies had reported it as reliable and valid^54^. The description in this subsection is mainly reproduced from our previous study^9^.

### General intelligence assessment

RAPMT was used to assess the general intelligence level^55^. RAPMT score was used to rule out the general intelligence effect on resting-state fMRI measures^32^. The test is performed to rule out the possible confounding factor from general intelligence in the association between resting-state functional connectivity and loneliness.

### Image acquisition

The imaging acquisition procedures mainly were reproduced from the previous study^53^. All MRI image acquisition was performed by using a 3-T Philips Achieva scanner. 160 functional volumes of axial gradient-echo images (64 × 64 matrix, 34 slices, voxel size: isotropic 3.75 mm, TR = 2000 ms, TE = 30 ms, flip angle = 70°, FOV = 24 cm) were acquired by using an echo-planar imaging (EPI) sequence for the resting-state fMRI analyses, which took 5 min 20 s. Participants were instructed to keep still with their eyes closed, not to sleep, and not to think anything particular. Pads and tapes were used to prevent movements of participants’ heads.

Prior to the resting-state fMRI scan, other functional images and diffusion-weighted data were acquired for the normalization of resting-state functional imaging data. The functional images were acquired from the N-back working memory task, performed before the structural scans. Diffusion-weighted data were acquired by using a spin-echo EPI sequence. Fractional anisotropy and mean diffusivity maps were constructed from the acquired diffusion-weighted images^56^. The description in this subsection is mainly reproduced from our previous study^52,53^.

### Preprocessing

Resting-state fMRI image preprocessing was performed using Data Processing Assistant for Resting-State fMRI (DPARSF), which is a part of the Data Processing and Analysis of Brain Imaging (DPABI) toolbox^57^ (http://rfmri.org/dpabi). DPARSF preprocessing is based on SPM12.

The skull of each participant’s first image was removed by masking the image using the signal threshold from the spatially smoothed BOLD images (8 mm FWHM). The skull removal procedure was performed so that these parts were not treated as the outer edge of the brain parenchyma in the pre-processing procedures. The functional images were slice timing corrected and realigned to fit the mean image of the series.

The rsfMRI functional Images were non-linearly registered to the N-back task functional image and coregistered to the diffusion-weighted images without gradient (*b* = 0). All functional images were subsequently normalized with voxel size isotropic 3.75 cm and using a validated two-step new segmentation algorithm and diffeomorphic anatomical registration of diffusion images through the exponentiated lie algebra-based registration process similar to the previous study^58^.

All 26 nuisance covariates, including Friston 24 motion parameters and the mean time-course of signals from the voxels within the white matter and CSF mask, were regressed from the functional images. The Friston 24-parameter model includes six head motion parameters, six head motion parameters (one-time point before), and the 12 corresponding squared items was used to regress head motion effects^59^. Additionally, the global signal was regressed out considering the merits, such as motion effect mitigation^31,60^. The processed images were spatially smoothed with 6-mm FWHM. After smoothing, the processed images were detrended and temporally band-pass filtered (0.01 < *f* < 0.08 Hz) to reduce low-frequency drift and high frequency. Seed-based functional connectivity analysis was performed after band-pass filtering. The description in this subsection is mostly reproduced from our previous study^52,53,61^.

### First-level analysis

Correlation maps were produced by extracting and computing the correlation coefficient of the BOLD time course of seed regions and that of all other brain voxels. This study examined the functional connectivity between all other brain voxels and four region-of-interest (ROI) seeds. Anterior insula, IFG, and TPJ are used as the seeds of the previous functional connectivity study^17^. Additionally, the DMPFC is also added as a seed region as it is related to social stimuli processing^14^. The examined seeds were all sphere-shaped with a radius of 6 mm. The coordinates of seed regions are shown in Table 4. All correlation values were transformed to Fisher’s Z-value for normalization purposes. The functional connectivity maps were acquired by subtracting the mean value within the whole-brain mask and dividing by the standard deviation of the whole-brain mask^62^.

**Table 4:** List of seed regions and their MNI coordinates.

Brain region		*x*	*y*	*z*
Anterior insula	R	40	16	4
Dorsomedial prefrontal cortex		6	54	21
Inferior frontal gyrus	R	54	2	0
Temporoparietal junction	R	68	− 22	24

*x*, *y*, *z*: coordinate in MNI.

### Second-level analysis

Second level statistical analysis, multiple regression, was applied to the Z-value of preprocessed images using SPM12 toolbox (https://www.fil.ion.ucl.ac.uk/spm/software/spm12/). The results were FWE corrected with *p* < 0.05 is classified as significant. The covariates used are age, sex, loneliness score, RAPMT score, and FD. FD Power score was used to rule out the head movement of the participants^63^. Multiple comparison correction using threshold-free cluster enhancement (TFCE) was applied to the statistical analysis^64^. Randomized nonparametric permutation testing (5000 permutations) was applied to the calculation by using TFCE toolbox (https://www.fil.ion.ucl.ac.uk/spm/ext/#TFCE). Permutation-based correction for multiple comparisons can be used to control the false positive rate of the brain data^65^.

## Supplementary Information

Supplementary Information.

## Data Availability

The datasets generated and/or analyzed during the current study are not publicly available due to the consent for the unrestricted release of participants’ personal information was not obtained from participants of this study. It is possible to provide data to third parties for purposes for which the subject has given consent (e.g., meta-analysis) or for which the ethics committee of Tohoku University, school of medicine has given consent. Therefore, any requests for reasons or formats that are not anticipated in advance must first be approved by the Ethics Committee of Tohoku University, school of medicine.
