# Supplementary material for: Loneliness inside of the brain: evidence from a large dataset of resting-state fMRI in young adult
Source: Sci Rep. 2022 May 12;12:7856. doi: 10.1038/s41598-022-11724-5 (PMC9098468; doi:10.1038/s41598-022-11724-5)
Supplement: Supplementary file 1 — Supplementary Information. [file 41598_2022_11724_MOESM1_ESM.pdf]

Loneliness Inside of the Brain: Evidence from Large Datasets of Resting-State fMRI in Young Adult

Denilson Brilliant T.[1,*], Hikaru Takeuchi[2], Rui Nouchi[3,4], Ryoichi Yokoyama[5], Yuka Kotozaki[6], Seishu Nakagawa[7,8], Sugiko Hanawa[7], Atsushi Sekiguchi[9], Shigeyuki Ikeda[10], Kohei Sakaki[1], Kelssy Hitomi dos Santos Kawata[11], Takayuki Nozawa[12], Susumu Yokota[13], Daniele Magistro[14], Ryuta Kawashima[1,2,3]

Affiliation:

[1] Department of Advanced Brain Science, Institute of Development, Aging and Cancer, Tohoku University, Sendai, Japan

[2] Division of Developmental Cognitive Neuroscience, Institute of Development, Aging and Cancer, Tohoku University, Sendai, Japan

[3] Smart-Aging Research Center, Tohoku University, Sendai, Japan

[4] Departments of Cognitive Health Science, Institute of Development, Aging and Cancer, Tohoku University, Sendai, Japan

[5] Suwa Red Cross Hospital, Suwa, Japan

[6] Division of Clinical Research, Medical-Industry Translational Research Center, Fukushima Medical University School of Medicine, Fukushima, Japan

[7] Department of Human Brain Science, Institute of Development, Aging and Cancer, Tohoku University, Sendai, Japan

[8] Division of Psychiatry, Tohoku Medical and Pharmaceutical University, Sendai, Japan

[9] Department of Behavioral Medicine, National Institute of Mental Health, National Center of Neurology and Psychiatry, Tokyo, Japan

[10] RIKEN Center for Advanced Intelligence Project, Tokyo, Japan

[11] Department of Mechanical and Intelligent Systems Engineering, The University of Electro-Communications, Tokyo, Japan

[12] Research Institute for the Earth Inclusive Sensing, Tokyo Institute of Technology, Tokyo, Japan

[13] Faculty of Arts and Science, Kyushu University, Fukuoka, Japan

[14] Department of Sport Science, Nottingham Trent University, Nottingham, England

* Author to whom correspondence should be addressed.

Supplementary Result

Multiple regression analysis between functional connectivity coefficient with DMPFC seed and loneliness score showed a trend of decreased functional connectivity. The functional connectivity between DMPFC and TPJ (x, y, z = -49, -45, 34, TFCE = 250.31; $p < .10$ FWE) are negatively correlated, albeit not significant, with loneliness score. The finding is shown in Fig S1.

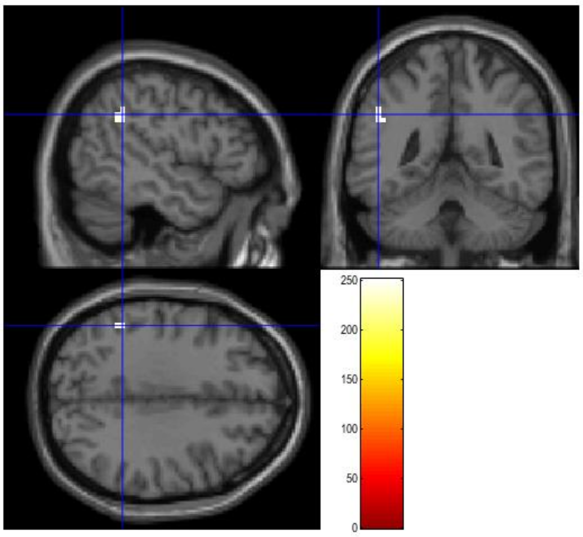

Supplementary Fig S1. Trend of negative effects of loneliness on resting-state functional connectivity with DMPFC as the seed region. The color represents the strength of the TFCE value. The result was obtained using the TFCE of $p < .10$ based on 5,000 permutations. The result was corrected within the left TPJ.

Supplementary Discussion

The functional connectivity between DMPFC and left TPJ tends to be negatively correlated with the loneliness score. DMPFC contributes to a variety of cognitive functions, such as conflict monitoring and decision making[1]. DMPFC also activates during viewing social-related features[2]. On the other hand, left TPJ is necessary to represent the theory of mind or believe others' beliefs[3]. Together, both areas play roles in the social mentalizing process[4]. The activity of both areas correlates with accuracy in predicting others' preferences[5]. Moreover, a previous study shows that functional connectivity between DMPFC and left TPJ is negatively correlated with punishing a group member that did a norm

violation[6]. The study suggested that the functional connectivity between DMPFC and left TPJ plays role in the mentalizing process to understand the reasons behind the norm violation[6]. Decreased functional connectivity between the two regions may suggest those lonely individuals have a problem with the theory-of-mind process that could lead to difficulty in inferring others' beliefs and understanding and tolerating the reason of norm violations.

Reference:

1. Venkatraman, V., Rosati, A. G., Taren, A. A. & Huettel, S. A. Resolving Response, Decision, and Strategic Control: Evidence for a Functional Topography in Dorsomedial Prefrontal Cortex. *Journal of Neuroscience* **29**, 13158–13164 (2009).
2. Wagner, D. D., Kelley, W. M., Haxby, J. V. & Heatherton, T. F. The Dorsal Medial Prefrontal Cortex Responds Preferentially to Social Interactions during Natural Viewing. *Journal of Neuroscience* **36**, 6917–6925 (2016).
3. Samson, D., Apperly, I. A., Chiavarino, C. & Humphreys, G. W. Left temporoparietal junction is necessary for representing someone else's belief. *Nat Neurosci* **7**, 499–500 (2004).
4. Wang, Y. *et al.* A large-scale structural and functional connectome of social mentalizing. *NeuroImage* **236**, 118115 (2021).
5. Kang, P., Lee, J., Sul, S. & Kim, H. Dorsomedial prefrontal cortex activity predicts the accuracy in estimating others' preferences. *Front. Hum. Neurosci.* **7**, (2013).
6. Baumgartner, T., Götte, L., Gügler, R. & Fehr, E. The mentalizing network orchestrates the impact of parochial altruism on social norm enforcement. *Hum. Brain Mapp.* **33**, 1452–1469 (2012).
